# Only consciousness truly exists? Two problems for IIT 4.0’s ontology

**DOI:** 10.3389/fpsyg.2024.1485433

**Published:** 2024-10-23

**Authors:** Ignacio Cea, Niccolo Negro, Camilo Miguel Signorelli

**Affiliations:** ^1^Center for Research, Innovation and Creation, and Faculty of Religious Sciences and Philosophy, Temuco Catholic University, Temuco, Chile; ^2^Department of Philosophy, Faculty of Philosophy and Humanities, Universidad Alberto Hurtado, Santiago, Chile; ^3^School of Psychological Sciences, Tel Aviv University, Tel Aviv, Israel; ^4^Department of Computer Science, University of Oxford, Oxford, United Kingdom; ^5^Center for Philosophy of Artificial Intelligence, University of Copenhagen, Copenhagen, Denmark; ^6^Laboratory of Neurophysiology and Movement Biomechanics, Université Libre de Bruxelles, Brussels, Belgium

**Keywords:** integrated information theory, intrinsicality problem, consciousness science, ontology of consciousness, formal metaphysics, intrinsic existence, extrinsic existence, idealism

## Abstract

In this article we present two ontological problems for the Integrated Information Theory of Consciousness 4.0: what we call the (i) the intrinsicality 2.0 problem, and (ii) the engineering problem. These problems entail that truly existing, conscious entities can depend on, and be engineered from, entities that do not objectively exist, which is problematic: if something does not exist in objective reality (i.e., in itself, independently of another entity’s consciousness), then it seems that it cannot be part of the material basis and determinants of other entities that do exist on their own. We argue that the core origin of these problems lies in IIT’s equation between true existence and phenomenal existence (consciousness), and the corresponding ontological exclusion of non-conscious physical entities (i.e., extrinsic entities) from objective reality. In short, these two problems seem to show that IIT should reconsider the ontological status of these extrinsic entities, because they need to exist objectively to account for the ontological implications of the scenarios we present here, which are permitted by the operational framework of the theory.

## 1 IIT 4.0: key scientific and ontological foundations

Currently, Integrated Information Theory (IIT) is recognized as one of the leading scientific theories of consciousness (Seth and Bayne, 2022; Ferrante et al., 2023; Signorelli et al., 2021). In contrast to other prominent theories, like the Global Neuronal Workspace Theory (Dehaene, 2014; Mashour et al., 2020; Dehaene et al., 2011), it primarily aims to explain the qualitative and subjective nature of experience, rather than targeting its neural, behavioral, computational, or functional correlates (Ellia et al., 2021; Albantakis et al., 2023). Methodologically, IIT is constructed from first principles, termed “axioms of phenomenal existence.” The most fundamental axiom is the irrefutable and immediately known fact that consciousness exists (the “zeroth axiom”). From this starting point, IIT then articulates the purportedly five essential properties of consciousness: intrinsicality, information, integration, exclusion, and composition. These are then translated into scientifically useful constructs, the “postulates of physical existence,” which turn the essential properties of experience into quantifiable, operational properties that define what it means for a physical system to be conscious. These postulates are mathematically formalized, and the “complex” (i.e., the physical substrate of consciousness) is identified, among overlapping systems, as the physical system that specifies the *maximal* value of *system integrated information* (*φ*_s_*), while “overlapping substrates with lower *φ_s_* are *excluded from existence*” (Albantakis et al., 2023, p. 12, italics added).

Importantly, this “exclusion from existence” is not a metaphor for IIT. It literally means that systems that do not specify *maximal φ_s_* (i.e., φ_s_*) do not truly exist. For IIT, only conscious entities truly exist because only they “exist for themselves,” absolutely rather than relatively (i.e., their existence is immediately and irrefutably known by the entities themselves) (Albantakis et al., 2023; Tononi et al., 2022). Hence, only *maximal*-*φ_s_* specifying complexes truly exist because only they exist consciously and hence “for themselves” in the relevant, phenomenal sense. In other words, if we were to list the entities that really exist in objective reality (i.e., in themselves, rather than just as part of another entity’s experience), we would need to count only those that exist subjectively, i.e., physical systems that exist intrinsically as subjective experiences (Cea et al., 2023).

This is the basis for IIT’s “great divide of being,” which is “the divide between what truly exists in an absolute sense, in and of itself—namely conscious, intrinsic entities—and what only exist in a relative sense, for something else” (Tononi et al., 2022, p. 8). In other words, only conscious intrinsic entities truly exist while non-conscious extrinsic entities, at best, only exist from the vicarious perspective of another intrinsic entity (e.g., as a unicorn “exists” when we imagine one), not in themselves (i.e., objectively, independently).

Thus, IIT is committed to what Cea et al. (2023) call the “principle of true existence,” according to which “only phenomenal existence is true existence” (p. 4). In consequence, IIT endorses an *eliminative* position towards, i.e., denies objective, independent existence to, all non-conscious physical entities that do not maximize *φ_s_*, including conventional macroscopic objects like our own bodies:

“Bodies and organs, tables and rocks, stars and planets…. are likely to unfold into extrinsic entities… They only exist vicariously, from the perspective of some intrinsic entity, and so *they do not truly exist*” (Tononi et al., 2022, p. 8, italics added).

In the following sections, we present two important problems that follow from this radical ontology: (i) the intrinsicality 2.0 problem, and (ii) the engineering problem. Both illustrate that IIT implies that truly existing conscious entities depend on, or can be engineered from, entities that do not objectively exist (i.e., extrinsic entities), which we take to be a significant theoretical issue suggesting that IIT should revise its ontological assumptions.

## 2 Two problems for IIT’s idealistic ontology

### 2.1 The intrinsicality problem 2.0

The intrinsicality problem 2.0 builds upon Mørch’s (2019) formulation of the intrinsicality problem for IIT, which exhibits a tension between the intrinsicality of consciousness and the requirement of *maximal φ_s_* which is, by definition, an extrinsic property (see also Fallon and Blackmon, 2021). Here, we develop this problem further in the context of IIT’s ontological elimination of extrinsic entities (hence the “2.0”). In short, the intrinsicality problem 2.0 states that, according to IIT, being a truly existing entity operationally depends on the properties of non-existent entities (i.e., ‘extrinsic entities’). This strikes us as a very problematic implication. Intuitively, the existence of intrinsic entities, such as a complex and associated experience, cannot depend on things that do not truly exist (i.e., that do not exist on their own, independently of another consciousness), but this is currently entailed by IIT’s ontological assumptions and formalism.

Let us explain in more detail. What we call “the intrinsicality problem 1.0” pointed out that specifying *maximal φ_s_* is by definition a relational, extrinsic property: the maximum value *in comparison to* the values of other (overlapping) candidate systems. This entails that changes in things outside the complex, either proximal or distant, can result in the complex being no longer a global maximum of *φ_s_* and hence ceasing to be conscious, even if internally it remains the same. Thus, claiming that consciousness is intrinsic would be problematic (Mørch, 2019). However, IIT proponents may reply that their conception of intrinsicality is not the standard philosophical one (Hendren et al., 2024a), according to which an intrinsic property of an object is a property whose instantiation is independent of anything external to that object (Lewis, 1983; Langton and Lewis, 1998). In contrast, IIT’s intrinsicality means that consciousness is “for itself”; it exists from the perspective of the system itself, which is operationalized in terms of intrinsic cause-effect power (i.e., “intrinsic information”). Thus, this meaning of intrinsicality in IIT is logically compatible with the operational dependence of the *φ_s_* measure on relational matters outside the complex.

However, what we call the “intrinsicality problem 2.0” is that things that do not specify maximal *φ_s_* do not exist on their own (i.e., “objectively”), and therefore, IIT entails that a complex, which enjoys intrinsic, absolute existence as a conscious entity, relationally depends on things (i.e., extrinsic entities) that do not truly exist (if these things do not also specify maximal *φ_s_* while not overlapping with the complex).

This is a troublesome implication. It means that, operationally, changing the properties of things that do not objectively exist can immediately determine whether another system truly exists or not. But how could extrinsic entities outside a complex, which purportedly exist only “operationally,” from the perspective of the experimenter’s consciousness, have such an effect on ontology, if they do not truly exist?

**Figure 1:**
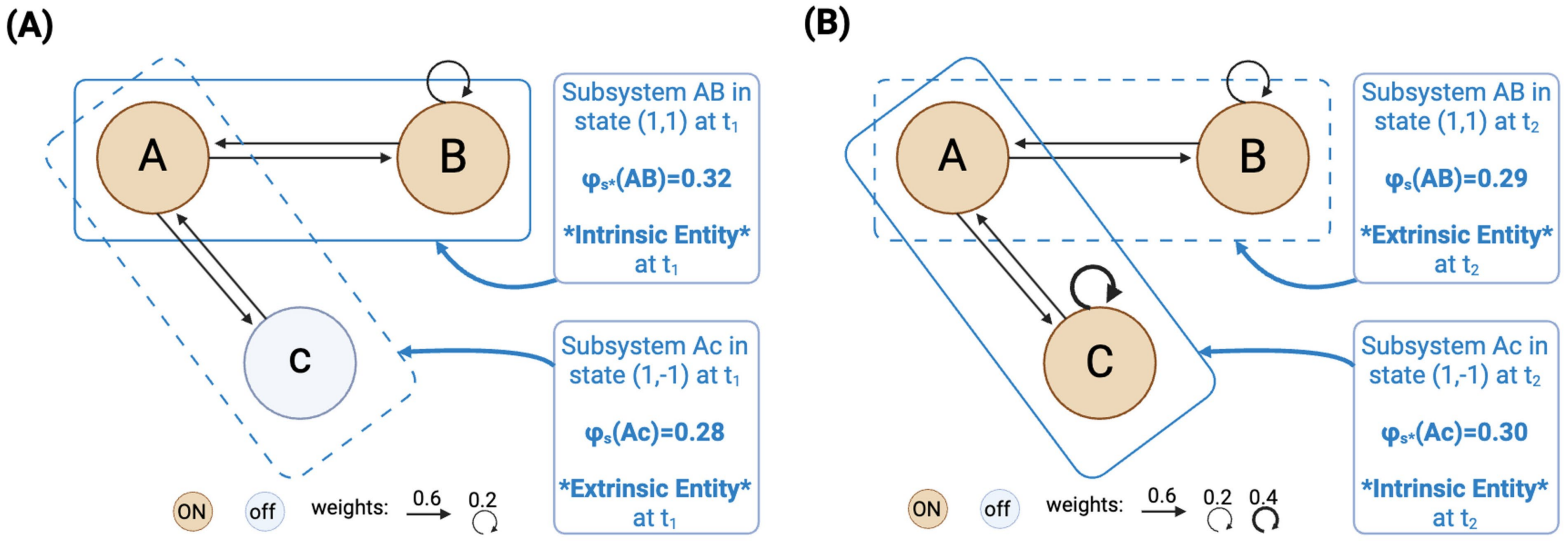
**(A)** A complex that is constituted by just two units: A and B interconnected and activated in a particular pattern such that it specifies the largest value of *φ_s_* among all overlapping subsets, and in particular, compared to a second subsystem AC that overlaps with the complex over unit A. **(B)** What previously was a truly existing intrinsic entity (Subsystem AB), turns into non-existence entirely due to modifications to a previously non-existing entity (unit C), which now becomes part of the new truly existing entity (Subsystem AC).

Consider Figure 1A, which illustrates a complex that is constituted by just two units: A and B, both active (1,1) at an initial time t_1_, linked by bidirectional excitatory connections weighted 0.6, plus a self-connection in B weighted 0.2. This AB subsystem overlaps with a second subsystem AC constituted by units A and C in state (1,−1) at time t_1_, also linked by bidirectional excitatory connections weighted 0.6. We computed the *φ_s_* values for each subsystem at t_1_.^1^ The results indicate that system AB specifies φ_s_* = 0.32, while system AC specifies *φ_s_* = 0.28. Hence, given IIT’s principle of maximal existence, i.e., “*what exists is what exists the most*” (Albantakis et al., 2023, p. 11), it means that only system AB is the complex at t_1_, while AC is “excluded from existence” (Albantakis et al., 2023, p. 12). In other words, at t_1_, only AB truly exists as an intrinsic, conscious entity, while AC only “exists,” at best, from the perspective of the intrinsic entity AB and the researcher analyzing both. Nor the subsystem constituted by the single unit “C” truly exists, because it specifies *φ_s_* = 0.

Now, the problem is that IIT’s operational framework allows the alteration of the properties of subsystem AC, which supposedly doesn’t truly exist, with severe consequences for AB. In particular, changing the properties of the extrinsic unit C can lead AB to lose its intrinsic existence, even if AB itself remains completely unchanged. Consider Figure 1B, it shows the results obtained by just turning unit C on, and adding to it a self-connection weighted 0.4, while keeping AB the same.^2^ These minor modifications determine that system AC at t_2_ specifies the new maximal *φ_s_* (φ_s_* = 0.30), while AB gets slightly behind (*φ_s_* = 0.29). Disconcertingly, this entails that the system that truly existed (AB), turns into non-existence entirely due to external, operational changes to the properties of the supposedly non-existing unit C (which was excluded from existence at t_1_), even if AB remains untouched. AC, in turn, comes into being as the new genuinely existing conscious entity thanks to purely operational and extrinsic modifications. Again, the fact that IIT entails that changes in non-existing things (extrinsic entities) have these dramatic ontological consequences seems very problematic: How is it that changes to, basically, mere ideas in consciousness (i.e., extrinsic entities) could determine that a truly existing conscious entity ceases to exist intrinsically? Isn’t it like ontologically eliminating an intrinsic entity just by thinking or imaging?

### 2.2 The engineering problem

What we call the “engineering problem” is also connected to the operational dependence of an intrinsic entity on extrinsic entities. Recall that for a given system to be conscious, its components must be interconnected and activated in such a way that the network specifies maximal system integrated information (φ_s_*). This is illustrated by Tononi’s assertion that “connecting first-order elements in certain ways is far from ontologically innocent, as it truly brings new things into being, including conscious beings” (Tononi, 2017, p. 632).

**Figure 2:**
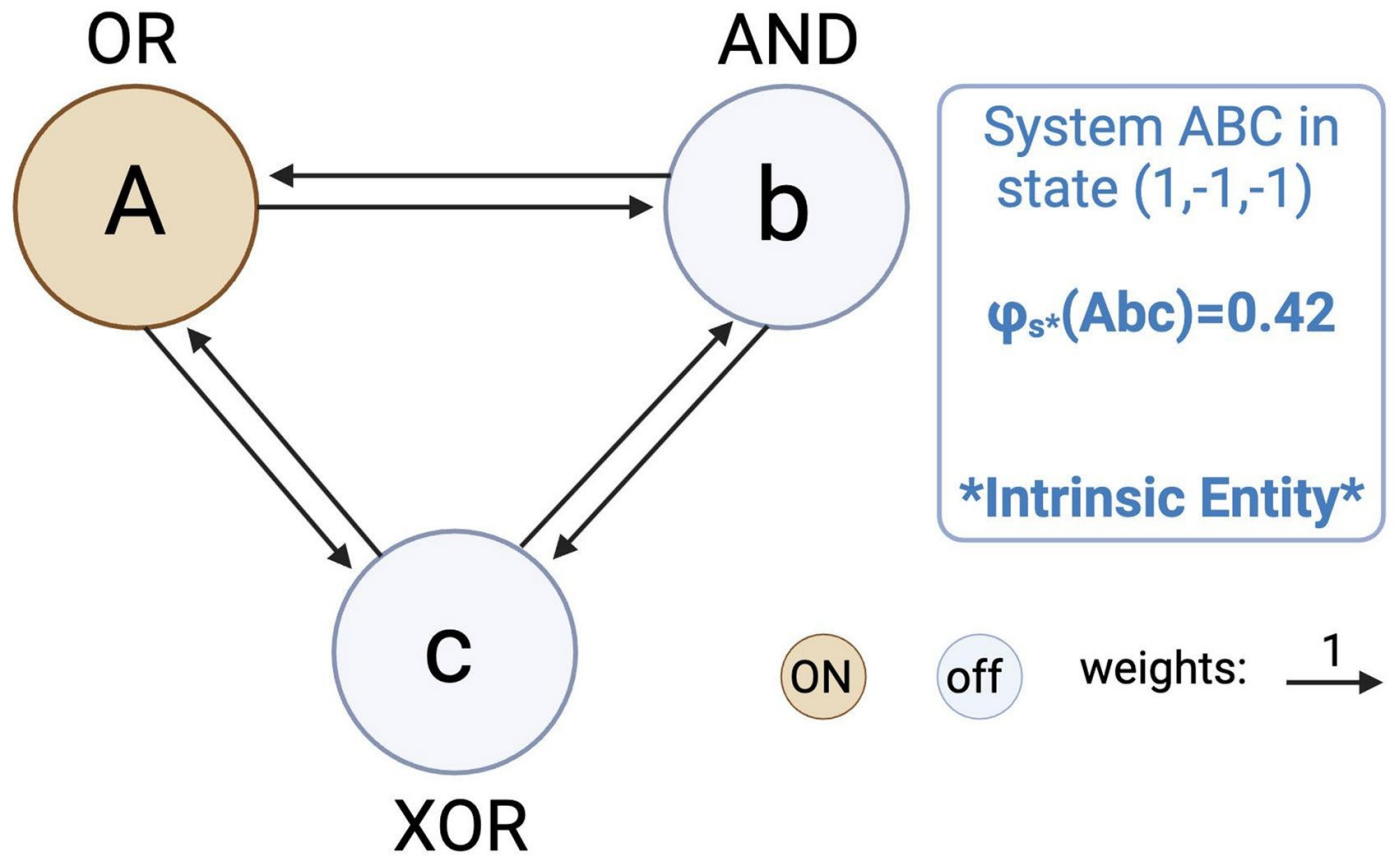
System ABC is constituted by three logic gates A = OR, B = AND, and C = XOR, in state (1,−1,−1), interconnected by bidirectional excitatory connections weighted 1. We found that this simple system specifies, as a whole, maximal system integrated information φ_s_* = 0.42. Hence, physically implementing this simple system in its current state means creating a truly existing conscious system out of previously nonexistent parts.

But the ontological implications of interconnecting units in such a way that a conscious, intrinsically existing system is created, seems to be possible only if the physical components (i.e., units and connections) exist in the first place: How could an engineer, following the tenets of IIT, build a conscious system out of non-existing physical parts? Here lies what we call the engineering problem. IIT entails that before constituting a system with maximal *φ_s_*, its components did not exist if they did not individually specify maximal *φ_s_*. At best, they only “existed” from the conscious perspective of some intrinsic entity. But this is exactly what seems to occur for simple systems like the one depicted in Figure 2. This network is constituted by three units, A = OR Gate; B = AND Gate; and C = XOR Gate, in state of activation (1,−1,−1). Again, we computed its system integrated information and found that ABC specifies a value of φ_s_* = 0.42. Hence, physically implementing this simple system in state (1,−1,−1) entails, according to IIT, engineering a truly existing conscious system.

As simple and naive as this seems, the problem is that the components of this system would presumably specify, individually, zero amount of maximal *φ_s_* before being assembled into the network. To build a concrete, physical implementation of that system, the engineer would just need to connect the three logic gates (OR, AND and XOR) on a breadboard, set the initial states to (1,−1,−1) using pull-up or pull-down resistors, interconnect them with a bunch of jumper wires, and power the circuit with a 5 V supply.^3^ But these components would presumably specify zero *φ_s_* on their own. Therefore, according to IIT, they would “exist,” at best, as extrinsic entities: only in the engineer’s consciousness, not in themselves.

For instance, the breadboard merely works as a passive platform to facilitate connections, without having the integrated structure and inner causal power needed to specify some *φ_s_* value (and the same can be said for the other components). Nonetheless, even if some rationale could be found to consider any of the components as a locus of *φ_s_* (e.g., a 5 V power adapter, due to its internally more complex structure), it seems highly unlikely that all of them do, and this is sufficient to support our claim: the engineer creates an intrinsic entity out of (maybe, some) nonexistent parts. More precisely, the engineer would create consciousness (i.e., intrinsic existence), from the arrangement of (at least, some) extrinsic entities that did not exist objectively (i.e., in themselves, independently of another entity’s consciousness). In short, IIT seems to entail that we can engineer an independent consciousness just by properly arranging ideas in our minds. We take this to be a very problematic implication.

## 3 Discussion

Our argumentation so far has shown that IIT implies that truly existing, intrinsic entities, depend on, and can be engineered from, extrinsic entities that do not objectively exist (i.e., in themselves, rather than just being part of another entity’s experience), which is problematic. To remedy this, IIT may consider revising its ontological assumptions. In particular, the problems we have presented suggest that extrinsic entities should not be excluded from objective existence, and consequently, that IIT’s great divide of being should be revised. A possible solution to address these cases seems to grant objective, independent existence to (at least some subset of) extrinsic entities, thereby rejecting both (i) IIT’s eliminativism towards (i.e., that denies objective, absolute existence to) unconscious systems that do not specify maximal *φ_s_*; and (ii) the associated principle of true existence, namely, that “only phenomenal existence is true existence” (Cea et al., 2023).

However, a first, potential reply from IIT may be called the ˝ontology-operationalization dissociation” objection. According to it, the problems rest on conflating the ontological and operational levels of IIT: intrinsic entities could depend their continuation on, and be engineered from non-existing extrinsic entities only from an operational point of view (i.e., third-person, instrumental, and inferential perspective), not ontologically (i.e., first-person, immediately known self-existence).

However, this reply leaves open the question of why the purely operational changes to the extrinsic system AC, specifically to its unit “C,” determine its coming into existence as a truly existent intrinsic entity, while the previous complex (system AB) becomes nonexistent (section 2.1.). How could a purportedly non-ontological, merely operational dependence of intrinsic entities on extrinsic entities have such an ontological import? In turn, how could assembling components that exist only from the third-person, operational point of view result in the coming into being of an intrinsic entity, if the manipulation of the components were “merely” operational (section 2.2.)? Thus, it seems that the operational and the ontological cannot be completely dissociated after all, because as our argumentation has shown, IIT entails that the right operational interventions have the power to affect the ontological status of the entities upon which we intervene. Also, this might suggest a potential mismatch between IIT’s ontological narrative and operational machinery (Signorelli et al., 2023).

A second, potential objection from IIT might build upon the theory’s concept of an “ontological dust” (Tononi et al., 2022). Accordingly, extrinsic entities may exist objectively after all, due to being reductively constituted by aggregates of “ontological dust”: minimally conscious intrinsic micro-entities that could –each one separately– specify maximal *φ_s_* and hence exist truly. Indeed, this is suggested by Tononi et al. (2022), who state that an unconscious body is “just an aggregate of much smaller [intrinsic] entities” (p. 8). Thus, a wire or an isolated logic gate may not specify maximal *φ_s_* as a whole, but in theory, could nonetheless be “condensed” (Albantakis et al., 2023, p. 19) into an exhaustive and non-overlapping set of intrinsic micro-entities (e.g., minimally conscious atoms). In that sense, IIT may reply, an extrinsic (macro) entity could exist objectively, although as nothing more than a collection of truly existing micro-entities.

In future work we critically address and reject this proposal (Cea et al., 2024b). The core of our argument is that IIT’s notion of an ontological dust entails the existence of an implausible type of entities: fundamental “monads” (Hendren et al., 2024b), i.e., partless, minimally conscious, intrinsic entities residing at the fundamental level of reality. We argue that fundamental monads are implausible because they contradict IIT’s own formalism and conceptualization regarding consciousness. That is, monads cannot specify maximal *φ_s_* and hence cannot qualify as (minimally) conscious intrinsic entities. This is because they are fundamentally partless, and no valid partition could be possibly exerted on them, which entails that the integration postulate cannot be coherently applied. Indeed, IIT itself stipulates that any candidate system must be susceptible of being divided “into k ≥ 2 parts” (Albantakis et al., 2023, p. 16), otherwise, it is not possible to run the equations needed to compute the *partitioned* transition probability matrices and associated *partitioned* cause/effect probabilities of the units of a candidate system (Eqs. 17 and 18, Albantakis et al., 2023, p. 17), which are, in turn, needed to compute *φ_s_* (Eqs. 19, 20 and 21, Albantakis et al., 2023, p. 17). In other words, monads violate what in future work we call IIT’s *plurality of parts/units requirement* to apply the integration postulate (Cea et al., 2024b). In forthcoming work we show that IIT also needs monads to account for the origin of consciousness, so their problematic status also affects IIT’s capacity to accommodate the phylogenetic evolution of subjective experience (Cea et al., 2024a). However, an intriguing possibility may be to consider monads not as partless elementary particles, but as ripples in a fundamental field, in line with the field integrated information hypothesis (Barrett, 2014). Another alternative is to grant objective existence to monads even if they are non-conscious, and consider consciousness/integrated information as an emergent property (Cea, 2021; Negro, 2022).

In sum, we take the problems we have presented as open problems for IIT that deserve further consideration. They suggest that IIT’s current ontology might be erroneously excluding extrinsic entities from objective reality, and that the equation “true existence = phenomenal existence” may need to be revised.

## Data Availability

The original contributions presented in the study are included in the article/supplementary material, further inquiries can be directed to the corresponding author.

## Footnotes

^1^All computations included in this article were performed using the “iit-4.0” new feature branch of the PyPhi toolbox available online as an interactive demo notebook based on Mayner et al. (2018): https://colab.research.google.com/github/wmayner/pyphi/blob/feature/iit-4.0/docs/examples/IIT_4.0_demo.ipynb.

^2^For example, in neuroscience turning on unit C and adding it a self-connection could mean that a neuron C was inactive at time t_1_ and then active at time t_2_ (where active may mean the activation of an action potential or firing), and that due to neuroplasticity anatomical properties of the neuron changed, making it in t_2_ more sensitive to firing and/or structurally different than at t_1_ (e.g., new anatomical connectivity, synaptic length, etc.) (Mateos-Aparicio and Rodríguez-Moreno, 2019; Puderbaugh and Emmady, 2023).

^3^Although IIT employs *models* of networks to apply its formalism, find results and derive conclusions about consciousness and its potential physical substrates; these models can, in principle, be physically *implemented* as actual circuits of interacting components. Thus, we take a model that according to IIT specifies some amount of *φ_s_** and hence consciousness, and specify what would be the concrete, practical requirements needed to actually implement that model as a real physical circuit.
